# Mortality and physical status at hospital discharge in Japanese elderly critically ill patients: a single-center retrospective study

**DOI:** 10.1186/s40981-017-0137-y

**Published:** 2018-01-03

**Authors:** Tomoaki Yatabe, Atsushi Nishigaki, Takahiko Tamura, Masataka Yokoyama

**Affiliations:** Department of Anesthesiology and Intensive Care Medicine, Kochi Medical School, Kohasu, Oko-cho, Nankoku, Kochi 783-8505 Japan

**Keywords:** Elderly patient, Intensive care, Outcome

## Abstract

**Background:**

Because there is ongoing population aging, the age of patients admitted to the intensive care unit (ICU) is also higher. However, the evidence about outcomes in elderly patients is insufficient in Japan. Therefore, we conducted a retrospective study.

**Method:**

The study participants were consecutive patients who were admitted to our ICU and received mechanical ventilation for more than 24 h. We divided the patients into two groups, according to age. Patients in group A were 74 years old or younger, and those in group B were 75 years old or older. The major outcome was in-hospital mortality.

**Findings:**

Two hundred and twenty patients met the inclusion criteria. There were 118 patients in group A and 102 patients in group B. The overall hospital mortality in both groups were similar (19 vs. 25%, *p* = 0.23). The proportion of patients who were discharged home and had good physical status at hospital discharge in group A were significantly higher than that in group B (72 vs. 37%, *p* < 0.0001; 91 vs. 74%, *p* = 0.004, respectively).

**Conclusion:**

The elderly population were associated with a twofold increase in the risk of discharged not to the home compared with others.

## Introduction

Because there is ongoing population aging in many countries, the median age of patients admitted to the intensive care unit (ICU) is also higher [1]. Thus, not only short- and long-term mortality, but also the quality of life after ICU discharge, is gaining attention from intensivists. A recent review described that although intensive care contributes to short-term survival among elderly critically ill patients, the 1-year mortality is 40–70% [1]. On the other hand, another study reported that short- and long-term mortality in elder patients decreased as same as younger patients for several years [2]. In addition, physical, cognitive, and functional disorders, as well as mental problems, referred to as post-intensive care syndrome (PICS) is an important issue in this field [3]. Of course, elderly critically ill patients face this problem [1]. In fact, only 25% of ICU survivors among patients aged 80 years or older returned to baseline physical function levels at 1 year after discharge from the ICU [4]. However, the evidence about outcomes in elderly patients is insufficient and almost all the epidemiological studies were performed in Europe, North America, and Oceania [1]. We thought that differences in health care systems such as medical insurance and number of medical providers influence the outcomes in elderly critically ill patients. Thus, the aim of this study was to assess in-hospital mortality and physical function at hospital discharge among our elderly ICU patients. Furthermore, we hypothesized that these outcomes in elderly patients might be worse compared with younger individuals. Therefore, we conducted a single-center retrospective study.

## Methods

The study was performed after approval by the ethics committee of Kochi Medical School Hospital (No. 28-139). The requirement for informed consent was waived considering the retrospective nature of the study. The study participants were consecutive patients who were admitted to our 12-bed capacity ICU and received mechanical ventilation for more than 24 h from April 2015 through March 2017.We excluded patients who were younger than 20 years, were not discharged by August 2017, were readmitted to our ICU during one hospital stay, and whose Acute Physiology and Chronic Health Evaluation (APACHE) II score could not be calculated due to insufficient data. We divided the patients into two groups, according to age. Patients in group A were 74 years old or younger at the time of admission to our ICU, and those in group B were 75 years old or older. Data regarding age, gender, height, weight, indication for ICU admission (surgical or non-surgical), APACHE II and the Sequential Organ Failure Assessment (SOFA) scores on the day of ICU admission, comorbidities (hypertension, diabetes mellitus, heart disease, cerebral stroke, and cancer), duration of mechanical ventilation, length of ICU and hospital stay, residence before hospitalization and discharge destination (home or other), physical status before ICU admission and at hospital discharge (walking, sitting, bed rest), and in-hospital mortality were collected from the medical records. We defined good physical status as walking and poor physical status as sitting and bed rest. The major outcome was in-hospital mortality, and secondary outcomes were the proportion of patients who were discharged home and good physical status at hospital discharge. Our hospital did not decide on standard criteria regarding discharge destination. Therefore, individual physicians decided on it based on patients’ statuses and request. We assessed each of these parameters using the non-paired *t* test, Mann–Whitney *U* test, and chi-square test. *p* values less than 0.05 were considered statistically significant. A receiver operating characteristic (ROC) curve was applied to determine the appropriate cutoff age for predicting good physical function at hospital discharge. In addition, we performed multivariable analysis regarding hospital mortality, physical function at hospital discharge, and discharge destination. Variables were considered potentially associated with the outcome when *p* < 0.05 in the univariate analysis. Statistical analysis was performed using JMP version 9.0 (SAS Institute Japan, Tokyo, Japan). Data were reported as mean ± standard deviation, median [interquartile range], or percentage.

## Results

Three hundred and seventy-seven patients received mechanical ventilation for more than 24 h during the study period. Of these patients, 15 who were younger than 20 years, 1 patient who was not discharged by August 2017, 13 patients who were readmitted to our ICU during one hospital stay, and 128 patients whose APACHE II scores could not be calculated were excluded. Thus, 220 patients met the inclusion criteria. There were 118 patients in group A and 102 patients in group B. The median age in both groups was 65 [55, 69] and 80 [77, 84] years (*p* < 0.0001) and the proportions of females were 36 and 40% (*p* = 0.48), respectively (Table 1). The proportion of surgical patients in group A and the APACHE II score in group B were significantly higher (74 vs. 59%, *p* = 0.02; 18 [13, 25] vs. 23 [17, 33], *p* < 0.0001, respectively). The duration of mechanical ventilation in group B was significantly longer than that in group A (3 [2, 5] vs. 4 [2, 8], *p* = 0.03). The length of ICU and hospital stay were not significantly different between the groups (5 [4, 9] vs. 7 [4, 10] days, *p* = 0.09; 36 [23, 55] vs. 39 [24, 58] days, *p* = 0.24, respectively). The overall hospital mortality in both groups were similar (19 vs. 25%, *p* = 0.23). In-hospital mortality among surgical and non-surgical patients were also not significantly different between the groups (8 vs. 13%, *p* = 0.30; 52 vs. 43%, *p* = 0.46, respectively). Thus, there were 95 hospital survivors in group A and 76 in group B. The proportion of patients who were discharged home and had good physical status at hospital discharge in group A were significantly higher than that in group B (72 vs. 37%, *p* < 0.0001; 91 vs. 74%, *p* = 0.004, respectively). The ROC curve analysis revealed that the best cutoff age for predicting physical function at hospital discharge was 81 years.

**Table 1:** Patient background and outcome data

	Group A	Group B	*p* value
	(< 74 years) (*N* = 118)	(≥ 75 years) (*N* = 102)	
Age, years	65 [55, 69]	80 [77, 84]	< 0.0001*
Gender (female), *N* (%)	42 (36)	41 (40)	0.48
Height, cm	162 ± 9	156 ± 9	< 0.0001*
Weight, kg	56 [48, 64]	51 [46, 57]	0.0008*
Body mass index, kg/m^2^	21.4 [18.8, 24.5]	21.3 [18.8, 23.5]	0.61
Reason of ICU admission			
Non-surgical, *N* (%)	31 (26)	42 (41)	0.02*
Surgical, *N* (%)	87 (74)	60 (59)	
Physical status before ICU admission			0.47
Good physical status, *N* (%)	96 (82)	79 (77)	
Poor physical status, *N* (%)	22 (18)	22 (23)	
Residence before hospitalization (home), *N* (%)	115 (97)	96 (94)	0.21
Comorbidity at hospitalization			
Hypertension, *N* (%)	56 (47)	62 (61)	0.048*
Diabetes mellitus, *N* (%)	37 (31)	23 (23)	0.14
Heart disease, *N* (%)	30 (25)	52 (51)	< 0.0001*
Cerebral stroke, *N* (%)	20 (17)	31 (30)	0.02*
Cancer, *N* (%)	53 (45)	24 (24)	0.009*
Acute kidney injury, *N* (%)	24 (20)	29 (28)	0.16
APACHE II	18 [13, 25]	23 [17, 33]	< 0.0001*
SOFA	6 ± 3	6 ± 3	0.20
Use of catecholamine on ICU admission day, *N* (%)	83 (70)	68 (67)	0.56
Use of renal replacement therapy, *N* (%)	24 (20)	22 (22)	0.82
Duration of ventilation days	3 [2, 5]	4 [2, 8]	0.03*
Length of ICU stay days	5 [4, 9]	7 [4, 10]	0.09
Length of hospital stay days	36 [23, 55]	39 [24, 58]	0.24
Hospital mortality, *N* (%)	23 (19)	26 (25)	0.29
Non-surgical, *N* (%)	16 (52)	18 (43)	0.46
Surgical, *N* (%)	7 (8)	8 (13)	0.30
Good physical status at hospital discharge, *N* (%)	86 (91)	56 (74)	0.004*
Discharge to home, *N* (%)	68 (72)	28 (37)	< 0.0001*

Mean ± standard deviation, median [interquartile range]

*ICU* intensive care unit, *APACHE II* Acute Physiology and Chronic Health Evaluation II, *SOFA* Sequential Organ Failure Assessment

*p <0.05

Hospital mortality rate in surgical patients was significantly lower than that in non-surgical patients in both groups A and B (8 vs. 52%, *p* < 0.0001; 13 vs. 43%, *p* < 0.0001, respectively) (Table 2). The proportion of patients who were discharged home and had good physical status at hospital discharge among surgical patients was significantly higher than that in non-surgical patients in both groups A and B. Multivariate analysis revealed that old age was not an independent predictor of in-hospital mortality (Table 3) Age, reason for ICU admission, residence before hospitalization, APACHE II score, and duration of ventilation were selected as candidate variables for multivariate analysis of physical status at hospital discharge (Table 4).These five variables and SOFA score were selected for the analysis of discharge destination. Younger age and shorter duration of ventilation were independent predictors of good physical status and discharge to the home (Table 3).

**Table 2:** Comparison of outcome data between non-surgical and surgical patients

A) Group A (< 74 years)			
	Surgery (*N* = 87)	Non-surgery (*N* = 31)	*p* value
Hospital mortality, *N* (%)	7 (8)	16 (52)	< 0.0001*
	*N* = 80	*N* = 15	
Good physical status at hospital discharge, *N* (%)	76 (95)	10 (67)	< 0.0001*
Discharge to home, *N* (%)	61 (76)	7 (47)	< 0.0001*
B) Group B (≥ 75 years)			
	Surgery (*N* = 60)	Non-surgery (*N* = 42)	*p* value
Hospital mortality, *N* (%)	8 (13)	18 (43)	< 0.0001*
	*N* = 52	*N* = 24	
Good physical status at hospital discharge, *N* (%)	42 (81)	14 (58)	0.04*
Discharge to home, *N* (%)	24 (46)	4 (17)	0.01*

*p <0.05

**Table 3:** Results of multivariate analysis

A) Hospital mortality			
	Reference	Odds ratio [95%CI]	*p* value
Age	< 74 years	1.02 [0.44–2.38]	0.96
Reason of ICU admission	Surgery	1.64 [0.60–4.39]	0.33
APACHE II	–	1.14 [1.08–1.21]	< 0.0001*
Hypertension	No	0.54 [0.24–1.21]	0.13
Heart disease	No	0.76 [0.32–1.76]	0.52
Cerebral stroke	No	0.73 [0.28–1.78]	0.50
Cancer	No	0.79 [0.31–2.03]	0.63
B) Physical status at hospital discharge			
	Reference	Odds ratio [95%CI]	*p* value
Age	–	1.05 [1.01–1.11]	0.02*
Reason of ICU admission	Surgery	1.77 [0.56–5.40]	0.33
Residence before hospitalization	Home	3.94 [0.32–95.1]	0.29
APACHE II	–	1.05 [0.98–1.13]	0.15
Duration of ventilation	–	1.13 [1.03–1.25]	0.009*
C) Discharge destination			
	Reference	Odds ratio [95%CI]	*p* value
Age	–	1.06 [1.03–1.10]	0.0002*
Reason of ICU admission	Surgery	2.57 [0.95–7.34]	0.06
Residence before hospitalization	Home	–	0.17
APACHE II	–	1.00 [0.94–1.09]	0.77
SOFA	–	1.04 [0.88–1.23]	0.65
Duration of ventilation	–	1.10 [1.01–1.24]	0.03*

*ICU* intensive care unit, *APACHE II* Acute Physiology and Chronic Health Evaluation II, *SOFA* Sequential Organ Failure Assessment

*p <0.05

**Table 4:** Univariate analysis of physical function at hospital discharge and discharge destination

A) Physical function at hospital discharge			
	Good (*N* = 142)	Poor (*N* = 29)	*p* value
Age, years	69.5 [64, 80]	82 [69.5, 87]	0.001*
Gender (female), *N* (%)	51 (36)	14 (48)	0.21
Body Mass Index, kg/m^2^	21.3 [18.9, 24.2]	20.2 [17.4, 23.8]	0.22
Reason of ICU admission			
Surgical, *N* (%)	118 (83)	14 (48)	0.0001*
Physical status before ICU admission			
Good physical status, *N* (%)	128 (90)	24 (83)	0.25
Residence before hospitalization (home), *N* (%)	141 (99)	26 (90)	0.001*
APACHE II	18 [14, 23]	25 [19, 34]	< 0.0001*
SOFA	5 ± 3	6 ± 3	0.06
Use of renal replacement therapy, *N* (%)	23 (16)	3 (10)	0.42
Duration of ventilation days	3 [2, 4]	6 [3. 10.5]	< 0.0001*
B) Discharge destination			
	Home (*N* = 96)	Other (*N* = 75)	*p* value
Age, years	68 [59, 75.8]	79 [69, 83]	< 0.0001*
Gender (female), *N* (%)	34 (35)	31 (41)	0.21
Body mass index, kg/m^2^	21.7 [19.3, 24.1]	20.6 [17.7, 24.1]	0.08
Reason of ICU admission			
Surgical, *N* (%)	85 (89)	47 (63)	< 0.0001*
Physical status before ICU admission			
Good physical status, *N* (%)	88 (92)	64 (85)	0.19
Residence before hospitalization (home), *N* (%)	96 (100)	71 (95)	0.02*
APACHE II	17 [14, 23]	22 [17, 27]	< 0.0001*
SOFA	5 ± 2	6 ± 3	0.01*
Use of renal replacement therapy, *N* (%)	11 (11)	15 (20)	0.12
Duration of ventilation days	3 [2, 4]	4 [2. 8]	0.0002*

Mean ± standard deviation, median [interquartile range]

*ICU* intensive care unit, *APACHE II* Acute Physiology and Chronic Health Evaluation II, *SOFA* Sequential Organ Failure Assessment

*p <0.05

## Discussion

We conducted the current retrospective study to reveal short-term mortality and physical status in elderly critically ill Japanese patients. We found that there were no significant differences in in-hospital mortality between elderly patients and younger patients. Multivariate analysis also supported this finding. However, physical status at hospital discharge in elderly patients was lower, and the elderly population was associated with a twofold increase in the risk of discharged not to home compared with others. A recent study described that the ICU and in-hospital mortality of patients aged 80 years or older were 1.5–2 times higher than patients younger than 80 years in the Netherlands (overall hospital mortality 11 vs. 21%) [2]. Another study reported that in-hospital mortality in patients aged ≥ 80 years was significantly higher compared with others (24 vs. 13%) and that age ≥ 80 years was associated with a 5.4-fold higher in-hospital mortality compared with younger age groups in Australia and New Zealand [5]. On the other hand, the overall in-hospital mortality in both groups was similar in our study. We compared patients aged 74 years or younger and patients aged 75 years or older. Although the definition of very old ICU patients was unclear, a recent study considered patients aged above 75–80 years as very old ICU patients [1]. In addition, the Japanese health care system defines the group above 75 years as being elderly. Therefore, we set 75 years as the cutoff age. In our data set, in-hospital mortality were not significantly different between individual aged 80 years or older and those younger than 80 years (25 vs. 15%, *p* = 0.08). In Japan, the short-term mortality in elderly critically-ill patients might not be higher compared with younger patients. Further multicenter studies are needed to confirm this result. The proportions of good physical status at hospital discharge and discharge to the home in group A were significantly higher than those in group B. Then, multivariable analysis also revealed that young age was an independent predictor of good physical status and discharge to the home. Review article about PICS reported that reduction of physical function may have an impact on the patient’s socioeconomic status and quality of life [3]. In addition, a recent study revealed that not being discharged to the home was an independent risk factor for moderate or severe disability at 6 months after ICU discharge [6]. In terms of PICS, differences of short-term physical function and discharge destination in elderly patients compared with the others were very important for intensivists.

Our study had several limitations. First, we could not discuss long-term mortality and physical function because we evaluated only in-hospital mortality. Therefore, long-term mortality such as 1-year mortality and long-term physical function in elderly patients might be worse than in younger patients. Second, our study was a single-center retrospective study. Thus, we could not be certain that our data represented all critically ill Japanese patients. We think that a multicenter observational study which evaluates not only short-term, but also long-term outcomes, is necessary.

## Conclusion

Our single-center retrospective study revealed that in-hospital mortality was similar between inpatients aged 75 years or older and those younger than 75 years among critically ill Japanese patients. However, in terms of physical status at hospital discharge, elderly patients were weaker, and the elderly population were associated with a twofold increase in the risk of discharged not to the home compared with others.
